# Non-Conventional Thermodynamics and Models of Gradient Elasticity

**DOI:** 10.3390/e20030179

**Published:** 2018-03-08

**Authors:** Hans-Dieter Alber, Carsten Broese, Charalampos Tsakmakis, Dimitri E. Beskos

**Affiliations:** 1 Faculty of Mathematics, Technische Universität Darmstadt, Schlossgartenstraße 7, D-64289 Darmstadt, Germany; 2 Department of Continuum Mechanics, Faculty of Civil Engineering, Technische Universität Darmstadt, Franziska-Braun-Str. 7, D-64287 Darmstadt, Germany; 3 Department of Civil Engineering, University of Patras, 26500 Patras, Greece

**Keywords:** gradient elasticity, non-equilibrium thermodynamics, interstitial working, boundary conditions, energy transfer law

## Abstract

We consider material bodies exhibiting a response function for free energy, which depends on both the strain and its gradient. Toupin–Mindlin’s gradient elasticity is characterized by Cauchy stress tensors, which are given by space-like Euler–Lagrange derivative of the free energy with respect to the strain. The present paper aims at developing a first version of gradient elasticity of non-Toupin–Mindlin’s type, i.e., a theory employing Cauchy stress tensors, which are not necessarily expressed as Euler–Lagrange derivatives. This is accomplished in the framework of non-conventional thermodynamics. A one-dimensional boundary value problem is solved in detail in order to illustrate the differences of the present theory with Toupin–Mindlin’s gradient elasticity theory.

## 1. Introduction

This paper addresses material bodies complying with a response function for free energy, which depends on both the strain tensor and its gradient. Usual gradient elasticity theories are of Toupin–Mindlin’s type (see Toupin [1], Mindlin [2] and Mindlin and Eshel [3]), i.e., they are characterized by response functions for Cauchy stress tensors, which obey the structure of a space-like Euler–Lagrange derivative of the free energy with respect to the strain tensor. (We use the term space-like Euler–Lagrange derivative with respect to some variable in the sense of Maugin [4]). In Broese et al. [5], a gradient elasticity model, referred to as Version 3, has been proposed for the first time, which is characterized by a classical constitutive law for the Cauchy stress at every material point in the interior of the body, i.e., the Cauchy stress is given by the usual derivative of the free energy with respect to the strain. Generally, one might ask, if it is possible to develop gradient elasticity theories of non-Toupin–Mindlin’s type, where the response function of the Cauchy stress tensor is not necessarily expressed in terms of space-like Euler–Lagrange derivative of the free energy with respect to the strain tensor. An affirmative answer to this question is of interest, for it encourages the development of theories which are more flexible than the usual ones, when modelling gradient effects. Actually, the elastic portion in the Korteweg’s fluid model is known to describe adequately experimental observed capillarity effects. However, the Cauchy stress in this model includes a term proportional to $grad^{2}\varrho$ (the second gradient of the mass density), which does not appear in the class of gradient elasticity models of Toupin–Mindlin’s type (cf. Dunn and Serrin [6], Auffray et al. [7] and Auffray et al. [8] and the references cited there). Evidently, every gradient elasticity theory should be consistent with the laws of thermodynamics. The aim of the paper is to indicate, in the framework of a non-conventional thermodynamics, that indeed gradient elasticity of non-Toupin–Mindlin’s type may be well formulated, i.e., consistent with the laws of thermodynamics.

In order to address non-localities over space and time, a non-conventional thermodynamics has been proposed in Alber et al. [9]. It has been shown in Broese et al. [5,10], that this thermodynamics provides a proper framework for discussing gradient elasticity models. In particular, the gradient elasticity model Version 3 has been established in the framework of this thermodynamics. It must be noted that limited motivation for the Version 3 model is provided in [5]. However, Version 3 as discussed in [5] should be regarded rather as a specific one-dimensional example and not as an exhaustive theory. In fact, the assumed boundary conditions are meaningful only for the discussed one-dimensional loadings. This is indicated on Section 5.2.2 in [5] where, before Equation (190), it is stated: “In the present paper we make the assumption ...”. Thus, the aim of the present paper is to highlight basic assumptions, which are necessary in order to establish rigorously the connection of Version 3 example with a sound constitutive theory. Others have stated that our effort aims to a first step towards developing gradient elasticity theories of non-Toupin–Mindlin’s type.

After some preliminaries in Section 2, mainly concerning the notation used in this work, we record in Section 3 the features of the assumed non-conventional thermodynamics. The main issue is that the hypothesis of the local equilibrium state of the usual irreversible thermodynamics is extended in order to capture non-local effects. This is achieved by using an energy transfer law in addition to the conventional energy balance law. The energy flux vector in this additional energy transfer resembles the interstitial work flux (cf. Dunn and Serrin [6] and Dunn [11]). Gradient elasticity theories in the framework of the non-conventional thermodynamics are discussed in Section 4. First, in Section 4.1, we present a fundamental constitutive structure for all gradient elasticity models we are interested in. Then, for reasons of comparison, we derive in Section 4.2 Toupin–Mindlin’s gradient elasticity theory as a trivial example of the assumed non-conventional thermodynamics. The gradient elasticity example of non-Toupin–Mindlin’s type, which has been called Version 3 in Broese et al. [5], is highlighted in Section 4.3 as a non-trivial example of the assumed thermodynamics. The main concerns in this section are focussed on the required boundary conditions. Section 4.4 deals with one-dimensional examples and especially provides a sound discussion of the one-dimensional example, which has been presented for the first time in Broese et al. [5]. The paper closes with a list of conclusions in Section 5.

## 2. Preliminaries

Throughout the paper, we use the same notation as in Broese et al. [5], which is essentially the same as in Mindlin [2] and Mindlin and Eshel [3], in order to facilitate comparison with these works. The deformations are assumed to be small, so we do not distinguish, as usually done, between reference and actual configuration. Unless explicitly stated, all indices will have the range of integers (1,2,3), with summation implied over repeated indices. All tensorial components are referred to a Cartesian coordinate system $\left\{x_{i}\right\}$ in the three-dimensional Euclidean point space, which induces the orthonormal basis $\left\{e_{i}\right\}$.

The Kronecker Delta is denoted by $\delta_{ij}$ and $A^{T}$ is the transpose of a second-order tensor **A**. For space and time derivatives we use the notations

(1) $$\partial_{i}\left(\right)\;:=\frac{\partial ()}{\partial x_{i}}={\left(\right)}_{,x_{i}}\;,\;{\left(\right)}^{\dot{}}=\frac{\partial ()}{\partial t}\equiv \frac{d()}{dt}\;,$$

where *t* is time. Explicit reference to space and time variables, upon which functions depend, will be dropped in most part of the paper. As often in physics, we find it convenient in several passages not to distinguish between functions and their values. However, to make things clear, when necessary, we shall give explicitly the set of variables which a function depends upon.

Let B be a material body, which may be identified by the position vectors $x=x_{i}e_{i}$. It is assumed that B is a single component system (no mixture), and that it occupies the space range V∪∂V in the three dimensional Euclidean point space we deal with, where *V* is an open manifold (the interior) with boundary ∂V. We denote by $n_{i}$ the components of the outward unit normal vector **n** to the surface ∂V bounding the space range *V*. For a function f(x,t), with x∈V∪∂V, the normal derivative Df and the surface derivative $D_{i}f$ are defined by

(2) $$Df\;:=n_{l}\partial_{l}f\;,$$

(3) $$D_{i}f=\partial_{i}f-n_{i}Df\;,$$

for every x∈∂V, (see Toupin [1] or Mindlin and Eshel [3]).

Let $u_{i}$ be the components of the displacement vector **u** and denote by $\varepsilon_{ij}$ and $k_{ijk}$ the components of the strain tensor ε and the gradient of ε, respectively:

(4) $$\varepsilon_{ij}=\frac{1}{2}\left(\partial_{i}u_{j}+\partial_{j}u_{i}\right)\;,$$

(5) $$k_{ijk}=\partial_{i}\varepsilon_{jk}=k_{ikj}\;.$$

Consider material bodies which are described by ordinary balance laws of linear and angular momentum, so that at any point in *V* the field equations

(6) $$\partial_{j}\Sigma_{jk}+F_{k}+I_{k}=0\;,$$

(7) $$\Sigma_{jk}=\Sigma_{kj}\;$$

apply. Here, $\Sigma_{kj}$ are the components of a Cauchy stress tensor Σ, $F_{k}$ are components of a body force vector **F** and $I_{k}$ are the components of an inertial force **I**. However, the two forces **F** and **I** may be composed respectively of classical and non-classical parts. If we define a generalized body force **b** through (cf. Gurtin et al. [12] (Section 19))

(8) b:=F+I

then, Equation (6) takes the form

(9) $$\partial_{j}\Sigma_{jk}+b_{k}=0\;.$$

It is perhaps of interest to make some comments on the generalized force **b**. In classical mechanics no parts of force **b** are present in boundary conditions. However, in gradient elasticity non-classical terms may be involved in force **b**, which can be assumed both to contribute or not to contribute to boundary conditions (see Mindlin [2] and Broese et al. [5]). Since, however, such aspects are not relevant to the present paper, we shall focus attention only on classical forces **b**, so that Equation (6) reads

(10) $$\partial_{j}\Sigma_{jk}+F_{k}-\varrho {\ddot{u}}_{k}=0\;,$$

with ϱ being the mass density and $F_{k}$ being components of classical body forces.

## 3. Non-Conventional Thermodynamic Framework

We intend to discuss gradient elasticity in the framework of the non-conventional thermodynamics proposed in Alber et al. [9]. In order to make the paper self-contained, we repeat here the general form of this thermodynamics.

Let *e* be the internal energy measured per unit volume, $w_{st}$ the stress power per unit volume and **q** the energy/heat flux vector (radiant heating is omitted). Toupin [1] suggested the possibility for **q** to encapsulate more than heat flux, and this has been in fact elaborated by Dunn and Serrin [6], and Dunn [11]. Then, the local form of the conventional energy balance for body B (first law of thermodynamics) reads

(11) $$\dot{e}=w_{st}-\partial_{i}q_{i}\;.$$

Fundamental in usual irreversible thermodynamics is the hypothesis of a local equilibrium state. It is assumed there that each material point of body B behaves like a simple homogeneous system in equilibrium, so that absolute temperature θ>0 and entropy per unit volume η may be assigned to that point. The free energy per unit volume is defined through

(12) ψ:=e−θη,

and the energy law (11) takes the equivalent form

(13) $$w_{st}-\dot{\psi }-\theta \dot{\eta }-\eta \dot{\theta }-\partial_{i}q_{i}=0\;.$$

The second law of thermodynamics is commonly accepted in the form of the Clausius– Duhem inequality

(14) $$\gamma \;:=\dot{\eta }+\partial_{i}\left(\frac{q_{i}}{\theta }\right)\;\geq \;0\;.$$

Now consider materials which are sensitive to non-localities in space and time effects. For example, assume the free energy function ψ to depend, besides on state variables permitted in classical irreversible thermodynamics, also on the spatial gradients of these variables. Since gradient terms indicate neighbourhood effects, the hypothesis of a classical local equilibrium state is generally no longer justified. Yet, according to classical irreversible thermodynamics, absolute temperature θ and entropy η can be attributed only to equilibrium states. We may proceed conceptually further along the lines of classical irreversible thermodynamics as follows.

The state of each material point of body B is assumed at any time to be associated with a *homogeneous material system in equilibrium*, which we call the *generalized associated local equilibrium state or generalized local accompanying state*. Classical thermostatics ensures for the generalized associated equilibrium system the existence of absolute temperature θ(x,t) and entropy η(x,t), and these are assumed to be the temperature and entropy of the real material at (x,t). Denote by $v_{I}$, $I=1,\cdots ,N_{I}$, the components of state variables and by $\xi_{J}$, $J=1,\cdots ,N_{J}$, the components of time and space derivatives of $v_{I}$, which are now assumed to enter into the response function of ψ for the real material,

(15) $$\psi =\psi (v_{I},\xi_{J},\theta )\;.$$

In contrast to Alber et al. [9], we do not include time and space derivatives of θ in $\xi_{J}$.

The mass density and the response function of free energy for the generally fictitious associated local equilibrium system are defined to be the same as for the real material characterized by Equation (15). For both, the real material and the associated equilibrium state, the free energy ψ and the internal energy *e* are postulated to satisfy Equation (12). In other words, not only the free energy, but also the internal energy is identical for the two systems. Let $w_{st}$ be the stress power and **q** the energy/heat flux vector for the real material, so that the energy balance laws (11) and (13) hold for the real material. We will introduce an energy balance for the generalized associated equilibrium system by regarding $\xi_{J}$ for this (homogeneous) system as new state variables, which are independent of $v_{I},\theta$. For example, assume $\varepsilon_{ij}$ and $\partial_{k}\varepsilon_{ij}$ to be included as state variables in the response function of ψ. Then, $\partial_{k}\varepsilon_{ij}$ have to be regarded for the generalized associated local equilibrium state as new, independent kinematical variables. These, again, engender additional, higher-order stresses and hence the stress power ${\bar{w}}_{st}$ entering into the energy balance law for the fictitious generalized associated local equilibrium state will be in general different from $w_{st}$. We denote by $\partial_{i}{\bar{q}}_{i}$ the energy/heat flux supply for the associated equilibrium system and postulate for this system the energy balance law

(16) $$\dot{e}={\bar{w}}_{st}-\partial_{i}{\bar{q}}_{i}\;\Leftrightarrow \;{\bar{w}}_{st}-\dot{\psi }-\theta \dot{\eta }-\eta \dot{\theta }-\partial_{i}{\bar{q}}_{i}=0\;.$$

Next, define stress power $w_{st}^{\prime }$ and energy/heat flux $q^{\prime }$ through

(17) $$\begin{array}{ccc} w_{st}^{\prime } & := & w_{st}-{\bar{w}}_{st}\;, \end{array}$$

(18) $$\begin{array}{ccc} q^{\prime } & := & q-\bar{q}\;, \end{array}$$

so that, from Equations (11) and (16),

(19) $$w_{st}^{\prime }=\partial_{i}q_{i}^{\prime }\;.$$

Further, assume that $w_{st}^{\prime }$ and $q^{\prime }$ can be decomposed in *N* parts $w_{st(i)}^{\prime }$ and $q_{\left(i\right)}^{\prime }$,

(20) $$\begin{array}{ccc} w_{st}^{\prime } & = & w_{st(1)}^{\prime }+w_{st(2)}^{\prime }+\cdots +w_{st(N)}^{\prime }\;, \end{array}$$

(21) $$\begin{array}{ccc} q^{\prime } & = & q_{\left(1\right)}^{\prime }+q_{\left(2\right)}^{\prime }+\cdots +q_{\left(N\right)}^{\prime }\;, \end{array}$$

and postulate energy / heat transfer into mechanical power through

(22) $$\begin{array}{ccc} w_{st(1)}^{\prime } & = & \partial_{i}q_{(1)i}^{\prime }\;,\\ w_{st(2)}^{\prime } & = & \partial_{i}q_{(2)i}^{\prime }\;,\\ & \vdots & \\ w_{st(N)}^{\prime } & = & \partial_{i}q_{(N)i}^{\prime }\;. \end{array}$$

In order to complete the theory, it remains to specify some constitutive equations for $w_{st(i)}^{\prime }$ and $q_{\left(i\right)}^{\prime }$. In doing so, it might be that new state variables will be involved.

The physical idea behind these equations is that, the energy/heat flux difference $q^{\prime }$, between the actual and the generalized local equilibrium state, may be composed of various parts, say *N*, which can be related to corresponding spatial interaction mechanisms of long range. These mechanisms provide the opportunity for producing some energy/heat transfer to mechanical power without affecting the internal energy, as manifested by Equations (19) and (22). The assumed transfer must be accounted for in the entropy production and hence it is postulated that

(23) $$\bar{\gamma }\;:=\dot{\eta }+\partial_{i}\left(\frac{{\bar{q}}_{i}}{\theta }\right)\geq 0\;,$$

or, in view of Equation (16),

(24) $$-\left(\eta \dot{\theta }+\dot{\psi }\right)+{\bar{w}}_{st}-\frac{1}{\theta }{\bar{q}}_{i}\partial_{i}\theta \geq 0\;.$$

Inequality (23) (respectively (24)) is the Clausius–Duhem inequality for the generalized associated local equilibrium state, which is supposed to apply for the real material as well. The concept of the generalized associated local equilibrium state imposes the existence of absolute temperature and entropy and motivates the introduction of inequality (23) or (24). Otherwise, these inequalities can be exploited by employing known methods in continuum thermodynamics. That means, like classical irreversible thermodynamics based on the hypothesis of a local equilibrium state, time and space derivatives of the state variables can be elaborated, when exploiting the inequality. Since it has been assumed in the present paper, that the response function of ψ does not depend on time and space derivatives of θ, we may use the Coleman-Noll procedure [13,14] to prove that

(25) $$\eta =-\frac{\partial \psi (v_{I},\xi_{J},\theta )}{\partial \theta }\;.$$

In conclusion, we would like to remark that the proposed thermodynamic approach is motivated by the interesting works of Dunn [11], Dunn and Serrin [6], Maugin [4,15], Toupin [1] and Ireman and Nguyen [16]. In particular, $q^{\prime }$ may be viewed as *interstitial work flux* (cf. Dunn and Serrin [6]). Under specific assumptions, theories based on the concept of non locality energy residual (see Polizzotto [17], Polizzotto [18], Liebe and Steinmann [19] and Liebe et al. [20]), and rested on the work of Edelen and Laws [21], are in line with the interstitial work flux approach. The particular feature of the non locality energy residual based theories is that they postulate a so-called insulation condition. The main difference to the aforementioned works is that $q^{\prime }$ in the present work is allowed to be given also implicitly as solution of differential equations, and not only explicitly as function of state variables. Additionally, no insulation condition is generally required. We shall turn back to these remarks at the end of Section 4.3.

## 4. Gradient Elasticity Theories in the Framework of the Non-Conventional Thermodynamics

### 4.1. Basic Constitutive Structure

We suppose that $w_{st}$ is given by

(26) $$w_{st}=\Sigma_{jk}{\dot{\varepsilon }}_{jk}\;,$$

with $\Sigma_{jk}$ introduced in Section 2, and that ψ is a function of ε,k and θ,

(27) ψ=ψ(x,t)=ψ(ε,k,θ).

Further, we assume that $w_{st}$ and **q** satisfy Equations (16)–(22), with N=1,

(28) $$w_{st}={\bar{w}}_{st}+w_{st}^{\prime }\;,\;q=\bar{q}+q^{\prime }\;,$$

and

(29) $$w_{st}-w_{st}^{\prime }-\dot{\psi }-\theta \dot{\eta }-\eta \dot{\theta }-\partial_{i}{\bar{q}}_{i}=0\;,$$

(30) $$w_{st}^{\prime }=\partial_{i}q_{i}^{\prime }\;.$$

By using Equations (25), (27) and the first of (28) in inequality (24), we obtain

(31) $$\begin{array}{c} w_{st}-w_{st}^{\prime }-\frac{\psi (\varepsilon ,k,\theta )}{\partial \varepsilon_{ij}}{\dot{\varepsilon }}_{ij}-\frac{\psi (\varepsilon ,k,\theta )}{\partial k_{ijk}}{\dot{k}}_{ijk}-\frac{1}{\theta }{\bar{q}}_{i}\partial_{i}\theta \;\geq \;0\;. \end{array}$$

With regard to the energy law (29), we define the fully recoverable isothermal case through θ = $\theta_{0}$ = const. and

(32) $$\theta_{0}\dot{\eta }+\partial_{i}{\bar{q}}_{i}=0\;,$$

(33) ψ=ψ(ε,k),

so that, from Equation (29),

(34) $$\begin{array}{c} w_{st}-w_{st}^{\prime }-\dot{\psi }=w_{st}-w_{st}^{\prime }-\frac{\psi (\varepsilon ,k)}{\partial \varepsilon_{ij}}{\dot{\varepsilon }}_{ij}-\frac{\psi (\varepsilon ,k)}{\partial k_{ijk}}{\dot{k}}_{ijk}=0\;. \end{array}$$

In this case, the inequality (31) reduces to an equality, namely Equation (34).

For definiteness, we set in the following (cf. Mindlin [2])

(35) $$\psi \left(x,t\right)=\psi \left(\varepsilon ,k\right)=\psi_{1}+\psi_{2}\;,$$

(36) $$\psi_{1}=\psi_{1}\left(x,t\right)=\psi_{1}\left(\varepsilon \right)=\frac{1}{2}\lambda \varepsilon_{ii}\varepsilon_{jj}+\mu \varepsilon_{ij}\varepsilon_{ij}\;,$$

(37) $$\psi_{2}=\psi_{2}\left(x,t\right)=\psi_{2}\left(k\right)=a_{1}k_{iik}k_{kjj}+a_{2}k_{ijj}k_{ikk}+a_{3}k_{iik}k_{jjk}+a_{4}k_{ijk}k_{ijk}+a_{5}k_{ijk}k_{kji}\;,$$

where λ, μ (Lamé constants) and $a_{1},\ldots ,a_{5}$ are material parameters. It is also convenient to define stresses τ and μ by

(38) $$\tau_{jk}\;\equiv \;\tau_{kj}\;:=\frac{\psi (\varepsilon ,k)}{\partial \varepsilon_{jk}}\;\equiv \;\frac{\psi_{1}\left(\varepsilon \right)}{\partial \varepsilon_{jk}}\;,$$

(39) $$\mu_{ijk}\;\equiv \;\mu_{ikj}\;:=\frac{\psi (\varepsilon ,k)}{\partial k_{ijk}}\;\equiv \;\frac{\psi_{2}\left(k\right)}{\partial k_{ijk}}\;.$$

Then, we deduce from Equation (34), with the help of (26), that

(40) $$\Sigma_{jk}{\dot{\varepsilon }}_{jk}-w_{st}^{\prime }-\tau_{jk}{\dot{\varepsilon }}_{jk}-\mu_{ijk}{\dot{k}}_{ijk}=0\;,$$

which is equivalent to

(41) $$\Sigma_{jk}{\dot{\varepsilon }}_{jk}-w_{st}^{\prime }=\dot{\psi }={\dot{\psi }}_{1}+{\dot{\psi }}_{2}\;,$$

and, in view of (30), equivalent to

(42) $$\Sigma_{jk}{\dot{\varepsilon }}_{jk}-\partial_{j}q_{j}^{\prime }=\dot{\psi }={\dot{\psi }}_{1}+{\dot{\psi }}_{2}\;.$$

According to Equation (41), the whole stress power $\bar{w}=\Sigma_{ij}{\dot{\varepsilon }}_{ij}-w_{st}^{\prime }$ will be stored in ψ (i.e., in the material). Equation (40), respectively Equation (41) or Equation (42), is a central equation for all gradient elasticity theories we shall discuss in the remainder of the paper. In order to obtain a global counterpart of Equation (42), we take the integral over *V* and apply the divergence theorem:

(43) $$\int_{V}\Sigma_{jk}{\dot{\varepsilon }}_{jk}dV-\int_{\partial V}n_{j}q_{j}^{\prime }dS=\frac{d}{dt}\int_{V}\psi dV\;.$$

The first integral on the left hand side can be recast as

(44) $$\begin{array}{ccc} \int_{V}\Sigma_{jk}{\dot{\varepsilon }}_{jk}dV & = & \int_{V}\Sigma_{jk}\left(\partial_{j}{\dot{u}}_{k}\right)dV\\ & = & \int_{\partial V}n_{j}\Sigma_{jk}{\dot{u}}_{k}dS+\int_{V}b_{k}{\dot{u}}_{k}dV\;. \end{array}$$

In deriving this formula, we multiplied the linear momentum Equation (9) with ${\dot{u}}_{k}$, integrated the result over *V* and applied the divergence theorem. After substitution of (44) into (43),

(45) $$\int_{\partial V}n_{j}\Sigma_{jk}{\dot{u}}_{k}dS-\int_{\partial V}n_{i}q_{i}^{\prime }dS+\int_{V}b_{k}{\dot{u}}_{k}dV=\frac{d}{dt}\int_{V}\psi dV\;,$$

which is a general balance law for ψ (see, e.g., Liu [22] (p. 31)).

To complete the formulation of gradient elasticity theories, we must specify $w_{st}^{\prime }$ and $q^{\prime }$ by suitable constitutive equations, which should be always compatible with the energy transfer law (30). In the two subsequent sections we consider two cases for such constitutive equations. In both cases we assume the response function of the Cauchy stress Σ to be of the form

(46) Σ=Σ(ε,k).

### 4.2. Toupin–Mindlin’s Type of Gradient Elasticity

We recover in this section Toupin–Mindlin’s gradient elasticity (see Toupin [1] and Mindlin [2]) by putting

(47) $$\begin{array}{ccc} w_{st}^{\prime } & = & -\left(\partial_{i}\mu_{ijk}\right){\dot{\varepsilon }}_{jk}-\mu_{ijk}{\dot{k}}_{ijk}\;, \end{array}$$

(48) $$\begin{array}{ccc} q_{i}^{\prime } & = & -\mu_{ijk}{\dot{\varepsilon }}_{jk}+c_{i}\;, \end{array}$$

where $c_{i}$ are the components of a divergence-free vector *c*,

(49) $$\partial_{i}c_{i}=0\;.$$

For simplicity, we set in the following c=0 and recall from Equation (5), that $\mu_{ijk}{\dot{k}}_{ijk}=\mu_{ijk}\partial_{i}{\dot{\varepsilon }}_{jk}$, so that, from Equations (47) and (48),

(50) $$w_{st}^{\prime }=-\partial_{i}\left(\mu_{ijk}{\dot{\varepsilon }}_{jk}\right)=\partial_{i}q_{i}^{\prime }\;.$$

In other words, the energy transfer law (30) is satisfied trivially (identically) for the case of the constitutive laws (47) and (48).

Next, on appealing to Equations (40) and (47),

(51) $$\Sigma_{jk}{\dot{\varepsilon }}_{jk}+\left(\partial_{i}\mu_{ijk}\right){\dot{\varepsilon }}_{jk}-\tau_{jk}{\dot{\varepsilon }}_{jk}=0\;,$$

or equivalently,

(52) $$\left(\Sigma_{jk}-\tau_{jk}+\partial_{i}\mu_{ijk}\right){\dot{\varepsilon }}_{jk}=0\;.$$

Bearing in mind Equations (38), (39) and (46), and by applying standard arguments, we deduce from Equation (52) that

(53) $$\Sigma_{jk}=\tau_{jk}-\partial_{i}\mu_{ijk}\;\equiv \;\frac{\partial \psi }{\partial \varepsilon_{jk}}-\partial_{i}\left(\frac{\partial \psi }{\partial \left(\partial_{i}\varepsilon_{jk}\right)}\right)\;.$$

The field Equation (9) (respectively (10)), together with the constitutive law (53) and appropriate initial and boundary conditions, make up Toupin–Mindlin’s gradient elasticity for classical body and inertial forces. It is worth noticing, that, with regard to Equation (53), the Cauchy stress tensor Σ is given by a space-like Euler–Lagrange derivative of ψ with respect to the strain tensor ε. This property is characteristic for the gradient elasticity models of the Toupin–Mindlin’s type.

The concomitant boundary conditions can be established by using Equation (48) with c=0 into Equation (45):

(54) $$\int_{\partial V}n_{j}\Sigma_{jk}{\dot{u}}_{k}dS+\int_{\partial V}n_{i}\mu_{ijk}\left(\partial_{j}{\dot{u}}_{k}\right)dS+\int_{V}b_{k}{\dot{u}}_{k}dV=\frac{d}{dt}\int_{V}\psi dV\;.$$

The surface integrals in this equation may be assembled in the manner indicated by Mindlin [2] (cf. also Broese et al. [5]):

(55) $$\int_{\partial V}n_{j}\Sigma_{jk}{\dot{u}}_{k}dS+\int_{\partial V}n_{i}\mu_{ijk}\left(\partial_{j}{\dot{u}}_{k}\right)dS=\int_{\partial V}\left[P_{k}{\dot{u}}_{k}+R_{k}\left(D{\dot{u}}_{k}\right)\right]dS\;,$$

where

(56) $$\begin{array}{ccc} P_{k} & := & n_{j}\Sigma_{jk}-D_{j}\left(n_{i}\mu_{ijk}\right)+\left(D_{l}n_{l}\right)n_{i}n_{j}\mu_{ijk}\;, \end{array}$$

(57) $$\begin{array}{ccc} R_{K} & := & n_{i}n_{j}\mu_{ijk}\;. \end{array}$$

Equation (55) suggests the boundary conditions

(58) $$\begin{array}{cc} \mathrm{either} & P_{k}\;\;\mathrm{or}\;\;u_{k} \end{array}$$

(59) $$\begin{array}{ccc} \mathrm{and} & \mathrm{either} & R_{k}\;\;\mathrm{or}\;\;Du_{k} \end{array}$$

have to be prescribed on ∂V. This completes the derivation of Toupin–Mindlin’s gradient elasticity as a trivial example of the adopted non-conventional thermodynamics.

Let us now assume that $q^{\prime }$ is the interstitial work flux. It can be recognized from Equation (48) that $q^{\prime }$ must be given as an explicit function of $\mu_{ijk}$ and ${\dot{\varepsilon }}_{jk}$, in order to derive Toupin–Mindlin’s gradient elasticity. In addition, the only Cauchy stress tensor appearing in the field equations (cf. Equation (9) or Equation (10)) is Σ, which justifies to denote Σ, given by the constitutive law (53), as the total Cauchy stress tensor for the Toupin–Mindlin’s theory. Then, the total Cauchy stress $\Sigma_{jk}$ is decomposed in the classical Cauchy stress $\tau_{jk}$ and the non-classical Cauchy stress $-\partial_{i}\mu_{ijk}$.

### 4.3. Gradient Elasticity of Non-Toupin–Mindlin’s Type

We start the construction of a gradient elasticity model of non-Toupin–Mindlin’s type by assuming $w_{st}^{\prime }$ to be given by the constitutive equation

(60) $$w_{st}^{\prime }=-{\dot{\psi }}_{2}\;\equiv \;-\mu_{ijk}{\dot{k}}_{ijk}\;,$$

and $q^{\prime }$ to be irrotational. Then, there exists a scalar-valued potential ϕ(x,t), so that

(61) $$q_{i}^{\prime }=\partial_{i}\phi \;.$$

It follows from Equations (30), (60) and (61), that ϕ fulfills the Poisson’s equation

(62) $$\Delta \phi \;:=\partial_{j}\partial_{j}\phi =\partial_{i}q_{i}^{\prime }=-{\dot{\psi }}_{2}=-\mu_{ijk}{\dot{k}}_{ijk}\;.$$

Its global counterpart is

(63) $$-\int_{\partial V}\left(D\phi \right)dS=\frac{d}{dt}\int_{V}\psi_{2}dV\;,$$

which is a general balance law for $\psi_{2}$.

Keeping in mind Equations (38), (46) and (60), we find from Equation (41) first that

(64) $$\Sigma_{jk}{\dot{\varepsilon }}_{jk}={\dot{\psi }}_{1}=\tau_{jk}{\dot{\varepsilon }}_{jk}\;,$$

and then, by employing standard arguments, that

(65) $$\Sigma_{jk}\;\equiv \;\tau_{jk}=\frac{\partial \psi_{1}\left(\varepsilon \right)}{\partial \varepsilon_{jk}}\;.$$

That means, the response function of the Cauchy stress Σ now does not exhibit the structure of a space like Euler–Lagrange derivative of ψ with respect to ε.

Next, by substituting (61) into (45),

(66) $$\int_{\partial V}n_{j}\Sigma_{jk}{\dot{u}}_{k}dS-\int_{\partial V}\left(D\phi \right)dS+\int_{V}b_{k}{\dot{u}}_{k}dV=\frac{d}{dt}\int_{V}\psi dV\;.$$

To proceed further, we have to specify Dϕ on ∂V. To this end, we assume the existence of a self-equilibrated Cauchy stress σ with components

(67) $$\sigma_{jk}=\sigma_{kj}\;;$$

i.e., σ satisfies in *V* the equilibrium equations

(68) $$\partial_{j}\sigma_{jk}=0\;.$$

In order to demonstrate the capabilities of the theory, which deals with small deformations, we shall concentrate ourselves in the remainder on problems which can be approximated by the specific Neumann boundary condition

(69) $$\begin{array}{ccc} -D\phi & = & n_{j}\sigma_{jk}{\dot{u}}_{k}\;\mathrm{on}\;\;\partial V\;. \end{array}$$

It is emphasized that Equation (69) holds only on ∂V. Of course, after integration over ∂V, and in view of Equations (63) and (68), we might infer from Equation (69) that

(70) $$\int_{V}\sigma_{jk}{\dot{\varepsilon }}_{jk}dV=\frac{d}{dt}\int_{V}\psi_{2}dV\;.$$

However, this is a global equation. In other words, we do not require from it to hold for arbitrary parts of the material body. To the contrary, Equation (63) is required to hold for arbitrary subbodies, which yields, by suitable continuity properties, the local Equation (62).

Turning back to Equation (66), and taking into account Equation (69), we find that

(71) $$\begin{array}{c} \int_{\partial V}n_{j}\left(\Sigma_{jk}+\sigma_{jk}\right){\dot{u}}_{k}dS+\int_{V}b_{k}{\dot{u}}_{k}dV=\frac{d}{dt}\int_{V}\psi dV\;. \end{array}$$

With respect to the surface integral in this equation, we define the surface traction

(72) $$P_{k}\;:=n_{j}T_{jk}$$

with

(73) $$T_{jk}\;:=\Sigma_{jk}+\sigma_{jk}$$

and impose the boundary conditions

(74) $$\begin{array}{cc} \mathrm{either} & P_{k}\;\;\mathrm{or}\;\;u_{k} \end{array}$$

have to be prescribed on ∂V. According to Equation (73), the stress **T** is decomposed in two parts Σ and σ, and since σ is self-equilibrated, the stress **T** satisfies also the balance law for linear momentum (cf. Equations (9) and (68))

(75) $$\partial_{j}T_{jk}+b_{k}=0$$

and the symmetry condition

(76) $$T_{jk}=T_{kj}\;.$$

The Equations (73), (75) and (76) justify to consider **T** as the total Cauchy stress tensor for the non-Toupin–Mindlin’s theory. In addition, according to Equations (61) and (69) the stress tensor σ causes on ∂V the energy flux $n_{i}q_{i}^{\prime }$. Once more it is outlined, that the boundary conditions must be compatible with Equations (63) and (69).

For the aims of the present paper it is not necessary to specify σ further. However, it is clear that the gradient elasticity we developed above deviates from gradient elasticities of Toupin–Mindlin’s type. Especially, the energy transfer law (30) is no more trivially satisfied (cf. Equation (62)), and neither of the Cauchy stresses Σ, σ and **T** is given by a space like Euler–Lagrange derivative of ψ with respect to ε. One can prove this by keeping in mind Equation (65), recalling Equations (73) and (68) and noting that neither the constitutive relation $\sigma_{jk}=\frac{\partial \psi }{\partial \varepsilon_{jk}}-\partial_{i}\left(\frac{\partial \psi }{\partial (\partial_{i}\varepsilon_{jk})}\right)$ nor the constitutive relation $\sigma_{jk}=-\partial_{i}\left(\frac{\partial \psi }{\partial (\partial_{i}\varepsilon_{jk})}\right)$ can hold generally. Otherwise the displacement field **u** should satisfy the field Equation (68) combined with any one of these constitutive laws and at the same time the field Equation (10) combined with the constitutive law (65). One-dimensional examples in the next section might help to verify this argument.

It is perhaps instructive to imagine ϕ as the negative rate of a variable φ, i.e.,

(77) $$\dot{\varphi }=-\phi \;,$$

and hence to rewrite Equation (61) in the form

(78) $$q_{i}^{\prime }=-\partial_{i}\dot{\varphi }\;,$$

and Equation (66) in the form

(79) $$\int_{\partial V}n_{j}\Sigma_{jk}{\dot{u}}_{k}dS+\int_{\partial V}\left(D\dot{\varphi }\right)dS+\int_{V}b_{k}{\dot{u}}_{k}dV=\frac{d}{dt}\int_{V}\psi dV\;.$$

The latter reflects the character of the power balance law in terms of rates and suggests how to incorporate variational formulations of the theory (cf. corresponding approaches in Auffray et al. [7] and Auffray et al. [8] and in the references cited there). After time integration of Equation (62), using suitable initial conditions,

(80) $$\Delta \varphi =\psi_{2}\;\geq \;0\;,$$

which indicates that φ is a subharmonic function. Equation (78) is analogous to Fourier’s heat conduction law, with φ and $\dot{\varphi }$ being thermal displacement and temperature like variables, respectively (cf. Green and Naghdi [23]), suggesting to incorporate φ as a new state variable. In the context of the present constitutive theory φ can be assumed to affect only the conductivity tensor in Fourier’s heat conduction law. A new, more elaborated constitutive theory might be formulated by assuming φ to enter into the response function of ψ as state variable. This aspect however is not pursued in the present paper. Essential features of the gradient elasticity developed in this section will be demonstrated in the next section, which provides a sound discussion on the one-dimensional example case presented for the first time in Broese et al. [5].

The considerations in this and the last section make clear that the proposed thermodynamics can be regarded as a generalization of the one according to Dunn and Serrin [6]. In fact, Dunn and Serrin [6] supposed $q^{\prime }$ to be given as an explicit function of state variables and their time and space derivatives. This was the case in Equation (48) for the Toupin–Mindlin’s gradient elasticity theory. However, apart from conceptual interpretations in conjunction with the generalized local equilibrium state, the adopted thermodynamics allows to express the interstitial work flux $q^{\prime }$ also as an implicitly defined functional of state variables, i.e., as solution of some differential equations like those in (61) and (62) for the non-Toupin–Mindlin’s gradient elasticity theory. This offers possibilities to capture more complex constitutive structures than the Toupin–Mindlin’s gradient elasticity.

### 4.4. One-Dimensional Examples

By substituting Equations (36) and (37) into Equations (38) and (39), we obtain

(81) $$\tau_{jk}=\lambda \delta_{jk}\varepsilon_{nn}+2\mu \varepsilon_{jk}\;,$$

(82) $$\mu_{ijk}=\frac{1}{2}a_{1}\left(\delta_{ij}k_{knn}+2\delta_{jk}k_{nni}+\delta_{ki}k_{jnn}\right)+2a_{2}\delta_{jk}k_{inn}+a_{3}\left(\delta_{ij}k_{nnk}+\delta_{ik}k_{nnj}\right)+2a_{4}k_{ijk}+a_{5}\left(k_{kij}+k_{jki}\right)\;.$$

Following Broese et al. [5], we set $x_{1}=x$ and consider a prismatic, slender bar of length *L* and cross section *A*, oriented along the *x*-axis. The only non vanishing component of the normal vector **n** along the *x*-axis is $n_{1}=\pm 1$. We put for the displacements $u_{i}$

(83) $$\begin{array}{c} u_{1}=u=u\left(x,t\right)\;, \end{array}$$

(84) $$\begin{array}{c} u_{2}=u_{3}\;\equiv \;0\;\mathrm{everywhere}\;, \end{array}$$

and for the material parameters in the elasticity law (81) we set λ=0, 2μ=E. With *E* denoting the classical Young’s modulus, we find that

(85) $$\begin{array}{c} \varepsilon \;:=\varepsilon_{11}=u_{,x}\;, \end{array}$$

(86) $$\begin{array}{c} k_{111}=\partial_{1}\varepsilon_{11}=\varepsilon_{,x}=u_{,xx}\;, \end{array}$$

(87) $$\begin{array}{c} \tau \;:=\tau_{11}=E\varepsilon =Eu_{,x}\;, \end{array}$$

(88) $$\begin{array}{c} \mu {}_{1}\;:=\mu_{111}=g^{2}E\;\varepsilon_{,x}=g^{2}E\;u_{,xx}\;, \end{array}$$

where

(89) $$g\;:=\sqrt{\frac{2}{E}\left(a_{1}+\cdots +a_{5}\right)}\;.$$

We intend to compare one-dimensional responses predicted by the developed gradient elasticity theory of non-Toupin–Mindlin’s type with those predicted by the Toupin–Mindlin’s theory. The governing equations for both theories are provided below. In both cases the body force **F** in Equation (10) is assumed to vanish.

#### 4.4.1. Non-Toupin–Mindlin’s Gradient Elasticity

From Equations (65) and (87) we see that

(90) $$\Sigma \;:=\Sigma_{11}=\tau =Eu_{,x}$$

and after inserting into Equation (10),

(91) $$\Sigma_{,x}-\varrho u_{,tt}=0\;.$$

Then, we set

(92) $$\sigma \;:=\sigma_{11}\;,$$

and assume that all the other components of the state variables vanish or play no role neither in the field equations nor in the boundary conditions. Since, from Equation (68),

(93) $$\sigma_{,x}=0\;,$$

we conclude that σ is a function only of time,

(94) σ=σ(t).

For the uniaxial problem here, the boundary conditions (74) are chosen in the form

(95) $$\begin{array}{ccc} u(0,t) & = & 0\; \end{array}$$

(96) $$\begin{array}{ccc} T(L,t) & = & \Sigma \left(L,t\right)+\sigma \left(t\right)=\frac{P_{0}}{A}e^{i\omega t}\;, \end{array}$$

where *i* is the imaginary unit. The given uniaxial load at the right hand side of Equation (96) is characterized by the operational frequency ω=const. and the amplitude of the axial load $P_{0}=const.$. By using (90) in (96),

(97) $$E\;u_{,x}\left(L,t\right)+\sigma \left(t\right)=\frac{P_{0}}{A}e^{i\omega t}\;.$$

Now, it is convenient to introduce the classical velocity of propagation of axial elastic waves

(98) $$c\;:=\sqrt{\frac{E}{\varrho }}\;,$$

the dimensionless variables

(99) $$\bar{x}\;:=\frac{x}{L}\;,\;\bar{t}\;:=\frac{c}{L}t\;,$$

the dimensionless parameters

(100) $$\bar{g}\;:=\frac{g}{L}\;,\;\bar{\omega }\;:=\frac{\omega L}{c}$$

and the dimensionless displacement and stresses

(101) $$\bar{u}\;:=\;\frac{u}{L}\;,\;{\bar{T}}_{0}\;:=\frac{P_{0}}{EA}\;,\;\bar{\Sigma }\;:=\frac{\Sigma }{E}\;,\;\bar{\sigma }\;:=\frac{\sigma }{E}\;,\;\bar{\mu }\;:=\frac{\mu {}_{1}}{EL}\;.$$

Thus, we may rewrite Equation (91) in the dimensionless form

(102) $${\bar{u}}_{,\bar{x}\bar{x}}-{\bar{u}}_{\bar{t}\bar{t}}=0\;,$$

while (94) becomes

(103) $$\bar{\sigma }=\bar{\sigma }\left(\bar{t}\right)\;.$$

The dimensionless form of the boundary conditions (95) and (97) reads

(104) $$\begin{array}{c} \bar{u}\left(0,\bar{t}\right)=0\;, \end{array}$$

(105) $$\begin{array}{c} {\bar{u}}_{,\bar{x}}\left(1,\bar{t}\right)+\bar{\sigma }\left(\bar{t}\right)={\bar{T}}_{0}e^{i\bar{\omega }\bar{t}}\;. \end{array}$$

Assuming the potential ϕ to be a function of x,t, i.e., ϕ=ϕ(x,t), we conclude from Equation (62) that

(106) $$\phi_{,xx}=-\mu {}_{1}u_{,xxt}=-g^{2}Eu_{,xx}u_{,xxt}\;.$$

In dimensionless form,

(107) $${\bar{\phi }}_{,\bar{x}\bar{x}}=-{\bar{g}}^{2}{\bar{u}}_{,\bar{x}\bar{x}}{\bar{u}}_{,\bar{x}\bar{x}\bar{t}}\;,$$

where the dimensionless potential $\bar{\phi }$ is defined by

(108) $$\bar{\phi }\;:=\frac{\phi }{cEL}\;.$$

The form of the loading conditions (104) and (105) suggests making the ansatz

(109) $$\bar{u}\left(\bar{x},\bar{t}\right)=\bar{U}\left(\bar{x}\right)e^{i\bar{\omega }\bar{t}}\;.$$

After inserting this ansatz into Equation (102), we arrive at the linear, ordinary differential equation with constant coefficients

(110) $${\bar{U}}_{,\bar{x}\bar{x}}+{\bar{\omega }}^{2}\bar{U}=0\;.$$

Its solution is

(111) $$\bar{U}=B\cos\left(\bar{\omega }\bar{x}\right)+C\sin\left(\bar{\omega }\bar{x}\right)\;.$$

The boundary condition (104) implies B=0, so that

(112) $$\bar{u}\left(\bar{x},\bar{t}\right)=Ce^{i\bar{\omega }\bar{t}}\sin\left(\bar{\omega }\bar{x}\right)\;.$$

By using this in Equation (107),

(113) $${\bar{\phi }}_{,\bar{x}\bar{x}}=-i{\bar{\omega }}^{5}{\bar{g}}^{2}C^{2}e^{2i\bar{\omega }\bar{t}}\sin^{2}\left(\bar{\omega }\bar{x}\right)\;.$$

Integration with respect to $\bar{x}$ between $\bar{x}=0$ and $\bar{x}=1$, furnishes

(114) $${\bar{\phi }}_{,\bar{x}}\left(1,\bar{t}\right)-{\bar{\phi }}_{,\bar{x}}\left(0,\bar{t}\right)=-i{\bar{\omega }}^{5}{\bar{g}}^{2}C^{2}e^{2i\bar{\omega }\bar{t}}\left[\frac{1}{2}-\frac{1}{4\bar{\omega }}\sin\left(2\bar{\omega }\right)\right]\;,$$

which corresponds to (63). Because of the boundary condition (104), the rate ${\bar{u}}_{,\bar{t}}$ vanishes at $\bar{x}=0$, i.e., ${\bar{u}}_{,\bar{t}}\left(0,\bar{t}\right)=0$, so that, from the boundary conditions (69)

(115) $$\begin{array}{ccc} \bar{x}=1 & : & {\bar{\phi }}_{,\bar{x}}\left(1,\bar{t}\right)=-\bar{\sigma }\left(\bar{t}\right){\bar{u}}_{,\bar{t}}\left(1,\bar{t}\right)\;, \end{array}$$

(116) $$\begin{array}{ccc} \bar{x}=0 & : & {\bar{\phi }}_{,\bar{x}}\left(0,\bar{t}\right)=0\;. \end{array}$$

From Equations (114)–(116),

(117) $$\bar{\sigma }{\bar{u}}_{,\bar{t}}\left(1,\bar{t}\right)=i{\bar{\omega }}^{5}{\bar{g}}^{2}C^{2}e^{2i\bar{\omega }\bar{t}}\left[\frac{1}{2}-\frac{1}{4\bar{\omega }}\sin\left(2\bar{\omega }\right)\right]\;,$$

and by appealing to Equation (112),

(118) $$\bar{\sigma }i\bar{\omega }Ce^{i\bar{\omega }\bar{t}}\sin\bar{\omega }=i{\bar{\omega }}^{5}{\bar{g}}^{2}C^{2}e^{2i\bar{\omega }\bar{t}}\left[\frac{1}{2}-\frac{1}{4\bar{\omega }}\sin\left(2\bar{\omega }\right)\right]\;.$$

Hence,

(119) $$\bar{\sigma }=\frac{{\bar{\omega }}^{4}{\bar{g}}^{2}C}{\sin\bar{\omega }}e^{i\bar{\omega }\bar{t}}\left[\frac{1}{2}-\frac{1}{4\bar{\omega }}\sin\left(2\bar{\omega }\right)\right]\;.$$

Finally, we use the latter and (112) in (105) to obtain

(120) $$C=\frac{{\bar{T}}_{0}}{\bar{\omega }\cos\bar{\omega }+\frac{{\bar{\omega }}^{4}{\bar{g}}^{2}}{\sin\bar{\omega }}\left[\frac{1}{2}-\frac{1}{4\bar{\omega }}\sin\left(2\bar{\omega }\right)\right]}\;,$$

which is the same result as in the Version 3 example in Broese et al. [5]. In particular, $-\Lambda \left(1,\bar{t}\right){\bar{u}}_{,\bar{x}}\left(1,\bar{t}\right)$ in [5] is equal to $\bar{\sigma }\left(\bar{t}\right)$ here. Once again, it is emphasized that the required boundary conditions in Version 3 example have been defined ad hoc in [5] and are valid only for the one dimensional loading case considered there. To the contrary, the required boundary conditions here are established by assuming in addition the existence of the self-equilibrated Cauchy stress tensor σ. In the calculated responses of $\bar{U}\left(\bar{x}\right)$ given below, ${\bar{T}}_{0}=0.0015$ has been chosen .

#### 4.4.2. Toupin–Mindlin’s Gradient Elasticity

From Equations (53), (87) and (88) we see that

(121) $$\Sigma \;:=\Sigma_{11}=\tau -\mu_{1,x}=E\left(u_{,x}-g^{2}u_{,xxx}\right)\;,$$

so that, after substitution into Equation (10),

(122) $$E\left(u_{,xx}-g^{2}u_{,xxxx}\right)-\varrho u_{,tt}=0\;.$$

It has been shown in Broese et al. [5] that the boundary conditions (56)–(59) reduce to

(123) $$\begin{array}{cc} \mathrm{either} & P\;:=\;P_{1}=n_{1}\left(\tau -\mu_{1,x}\right)=n_{1}E\left(u_{,x}-g^{2}u_{,xxx}\right)\;\mathrm{or}\;u \end{array}$$

(124) $$\begin{array}{cc} \mathrm{and}\;\mathrm{either} & R\;:=\;R_{1}=\mu_{1}=g^{2}Eu_{,xx}\;\mathrm{or}\;n_{1}u_{,x} \end{array}$$

have to be prescribed at x=0 and x=L. If we assume $u_{,xx}\left(0,t\right)=0$, and $u_{,x}\left(L,t\right)=\varepsilon_{0}e^{i\omega t}$, with $\varepsilon_{0}$ being a prescribed strain value, then the one-dimensional boundary conditions corresponding to those in Equations (95) and (96) read

(125) $$\begin{array}{c} u\left(0,t\right)=0\;,\;u_{,xx}\left(0,t\right)=0\;,\;u_{,x}\left(L,t\right)=\varepsilon_{0}e^{i\omega t}\;, \end{array}$$

(126) $$\begin{array}{c} \tau \left(L,t\right)-\mu_{1,x}\left(L,t\right)=E\left[u_{,x}\left(L,t\right)-g^{2}u_{,xxx}\left(L,t\right)\right]=\frac{P_{0}}{A}e^{i\omega t}\;. \end{array}$$

By employing the dimensionless variables introduced in Equations (99)–(101), we can recast the field Equation (122) and the boundary conditions (125) and (126) as follows

(127) $$\begin{array}{c} {\bar{u}}_{,\bar{x}\bar{x}}-{\bar{g}}^{2}{\bar{u}}_{,\bar{x}\bar{x}\bar{x}\bar{x}}-{\bar{u}}_{\bar{t}\bar{t}}=0\;, \end{array}$$

(128) $$\begin{array}{c} \bar{u}\left(0,\bar{t}\right)=0\;,\;{\bar{u}}_{,\bar{x}\bar{x}}\left(0,\bar{t}\right)=0\;,\;{\bar{u}}_{,\bar{x}}\left(1,\bar{t}\right)=\varepsilon_{0}e^{i\bar{\omega }\bar{t}}\;, \end{array}$$

(129) $$\begin{array}{c} {\bar{u}}_{,\bar{x}}\left(1,\bar{t}\right)-{\bar{g}}^{2}{\bar{u}}_{,\bar{x}\bar{x}\bar{x}\bar{x}}\left(1,\bar{t}\right)={\bar{T}}_{0}e^{i\bar{\omega }\bar{t}}\;. \end{array}$$

The form of the loading condition (129) suggests making the same ansatz as in Equation (109):

(130) $$\bar{u}\left(\bar{x},\bar{t}\right)=\bar{U}\left(\bar{x}\right)e^{i\bar{\omega }\bar{t}}\;.$$

The problem (127)–(129) has been solved in Broese et al. [5], by means of the ansatz (130). The solution reads

(131) $$\bar{U}\left(\bar{x}\right)=C_{1}e^{\xi_{1}\bar{x}}+C_{2}e^{-\xi_{1}\bar{x}}+C_{3}cos\left(\xi_{2}\bar{x}\right)+C_{4}sin\left(\xi_{2}\bar{x}\right)\;,$$

where $\pm \xi_{1}$ and $\pm i\xi_{2}$ are the eigenvalues of the differential Equation (127). The constants $C_{1},C_{2},C_{3},C_{4}$ have been determined in [5] from the boundary conditions (128) and (129) as functions of $\xi_{1}$ and $\xi_{2}$. In the calculated responses of $\bar{U}\left(\bar{x}\right)$ given below we have chosen ${\bar{T}}_{0}=0.0015$ as above and ${\bar{U}}_{,\bar{x}}\left(1\right)=0.0075$ in the second of the boundary conditions in (128).

Classical elasticity is recovered from Equation (127) for $\bar{g}=0$. For this case,

(132) $${\bar{u}}_{,\bar{x}\bar{x}}-{\bar{u}}_{,\bar{t}\bar{t}}=0\;,$$

and by employing the ansatz (130),

(133) $${\bar{U}}_{,\bar{x}\bar{x}}+{\bar{\omega }}^{2}\bar{U}=0\;,$$

which has the solution

(134) $$\bar{U}=B\cos\left(\bar{\omega }\bar{x}\right)+C\sin\left(\bar{\omega }\bar{x}\right)\;.$$

The first of the boundary conditions in (128) yields B=0. Note that the second boundary condition in (128) is non-classical and therefore it is not required to hold for classical elasticity. The boundary condition (129) for $\bar{g}=0$ is a classical one and furnishes $C=\frac{{\bar{T}}_{0}}{\bar{\omega }\cos\bar{\omega }}$. Hence, for classical elasticity $\bar{U}\left(\bar{x}\right)$ takes the form

(135) $$\bar{U}\left(\bar{x}\right)=\frac{{\bar{T}}_{0}}{\bar{\omega }\cos\bar{\omega }}\sin\left(\bar{\omega }\bar{x}\right)\;.$$

As above, ${\bar{T}}_{0}=0.0015$ has been chosen in the calculated responses in the next section.

#### 4.4.3. Comparison of the Two Theories with Reference to the One-Dimensional Loading Case

The gradient elasticity theories of non-Toupin–Mindlin’s type and of Toupin–Mindlin are different in both the field equations and the boundary conditions. Especially, with regard to the derivatives with respect to $\bar{x}$, the differential equation governing the response of the displacement $\bar{u}$ is of second-order for the non-Toupin–Mindlin’s theory (see Equation (102)) and of fourth-order for the Toupin–Mindlin’s theory (see Equation (127)). Further, all boundary conditions in Equations (95) and (96) for the non-Toupin–Mindlin’s theory are classical. To the contrary, not all boundary conditions in Equations (125) and (126) for the Toupin–Mindlin’s theory are classical. Gradient effects are controlled in both theories by the parameter $\bar{g}$ (respectively *g*). For the case of the Toupin–Mindlin’s theory this parameter is present in the constitutive law for the stress Σ (see Equation (121)) as well as in the boundary conditions (cf. Equations (127) and (129)). To the contrary, in the case of the non-Toupin–Mindlin’s theory this parameter is present only in the boundary condition (105) and not in the constitutive law (90) for Σ. Actually, the boundary condition (105) depends on the stress $\bar{\sigma }$ and this in turn depends on $\bar{g}$, as it is shown in Equation (119). In other words, $\bar{\sigma }$ affects the response of $\bar{u}$ only by the boundary condition (105).

**Figure 1:**
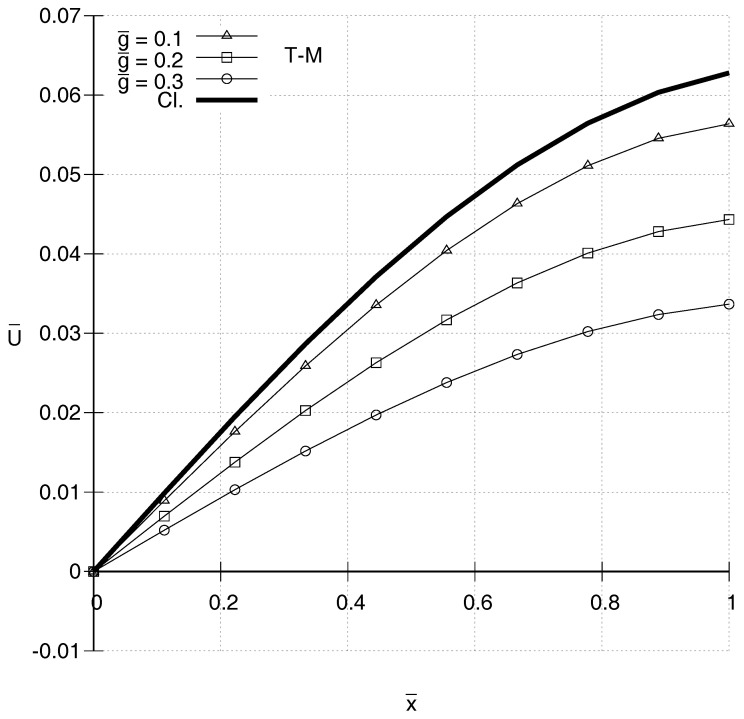
Predicted distributions according to the Toupin–Mindlin’s theory (T–M) for $\bar{\omega }=1.4$ and various values of $\bar{g}$.

**Figure 2:**
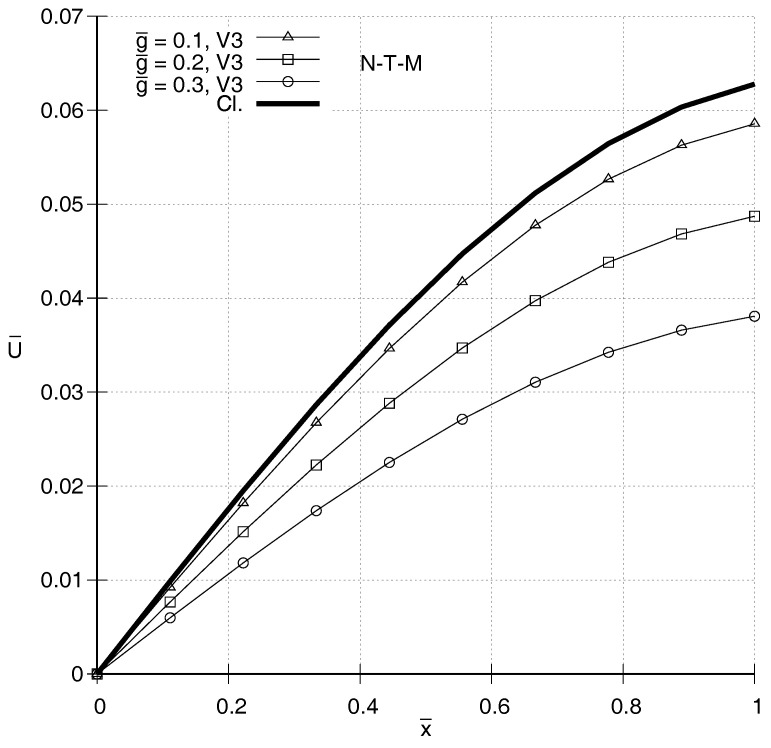
Predicted distributions according to the non-Toupin–Mindlin’s theory (N-T–M) for $\bar{\omega }=1.4$ and various values of $\bar{g}$. The distributions are qualitatively similar to those in Figure 1.

**Figure 3:**
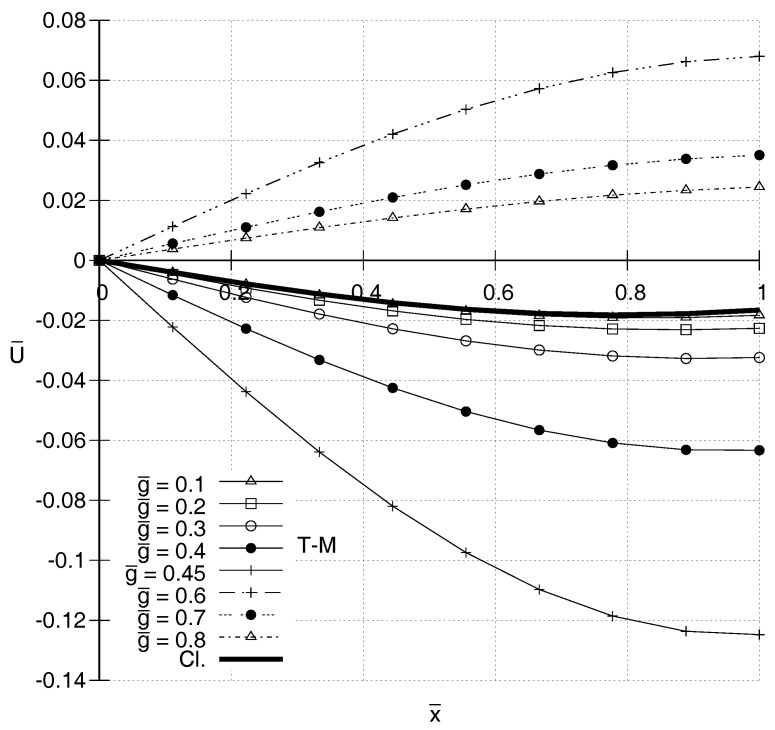
Predicted distributions according to the Toupin–Mindlin’s theory (T–M) for $\bar{\omega }=2.0$ and various values of $\bar{g}$.

**Figure 4:**
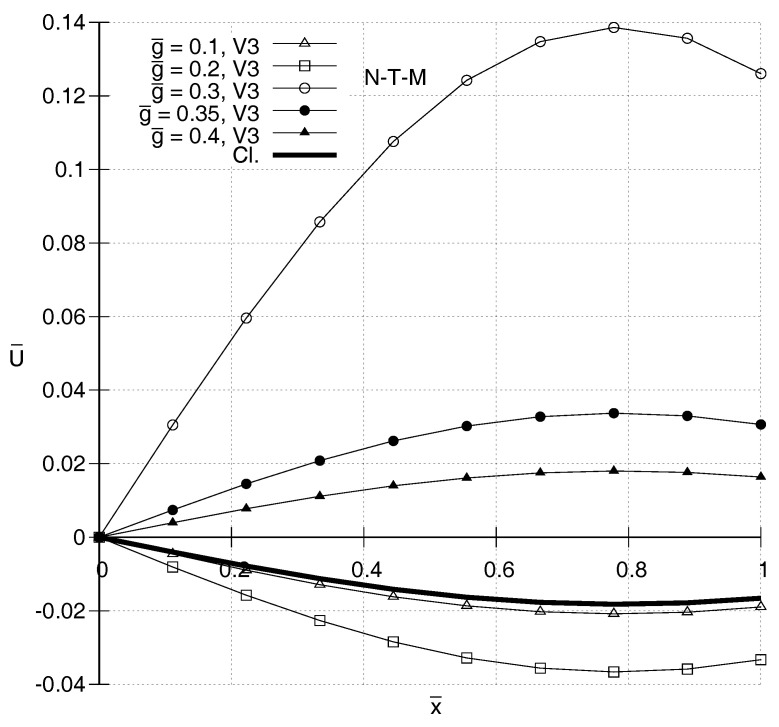
Predicted distributions according to the non-Toupin–Mindlin’s theory (N-T–M) for $\bar{\omega }=2.0$ and various values of $\bar{g}$. The distributions are qualitatively similar to those in Figure 3.

It is also of interest to compare distributions of $\bar{U}\left(\bar{x}\right)$ according to the two gradient elasticity theories with each other and with the one predicted by classical elasticity. In Figure 1, Figure 2, Figure 3 and Figure 4, N-T–M, T–M and Cl. denote the responses according to the gradient elasticity of the non-Toupin–Mindlin’s type, the gradient elasticity of Toupin–Mindlin and the Classical elasticity, respectively. It can be seen in Figure 1 and Figure 2 that, for $\bar{\omega }=1.4$, the distributions $\bar{U}\left(\bar{x}\right)$ for both gradient elasticity theories tend to be closer to the classical one, the smaller the value of $\bar{g}$. These figures indicate qualitatively similar responses for the two theories. This holds true also for higher frequencies as shown in Figure 3 and Figure 4. For low frequencies, as in Figure 1 and Figure 2, a stiffening effect is observed, i.e., decreasing absolute values of $\bar{U}$ with increasing values of $\bar{g}$. However, the opposite happens for $\bar{\omega }=2.0$ in Figure 3 and Figure 4, and generally the stiffening effect for both gradient elasticities is dependent on the frequency $\bar{\omega }$.

## 5. Conclusions

1. We adopted in the paper a non-conventional thermodynamics framework, which allows to address gradient elasticity models. The main issue in this thermodynamics is the assumption of an energy transfer equation in addition to the usual energy balance law. This is equivalent to allowing the interstitial work flux to be defined not only as an explicit function of state variables but also as an implicitly defined functional of state variables.
2. Toupin–Mindlin’s gradient elasticity is derived by satisfying the additional energy transfer equation identically.
3. A gradient elasticity model of non-Toupin–Mindlin’s type is established as a non-trivial example of the assumed energy transfer law.
4. The required boundary conditions in the model of non-Toupin–Mindlin’s type are established by employing a self-equilibrated stress tensor as part of the total Cauchy stress tensor at the boundary. This allows to provide a rigorous justification of the ad hoc assumptions in the Version 3 example studied earlier.
5. The most important conclusion of the paper is that we indicated a possibility for modelling gradient elasticity of non-Toupin–Mindlin’s type. This result suggests pursuing further the ideas introduced in the paper when modeling gradient effects.

## References

[1] Toupin R. (1962). Elastic materials with couple-stresses. Arch. Ration. Mech. Anal..

[2] Mindlin R. (1964). Micro-structure in linear elasticity. Arch. Ration. Mech. Anal..

[3] Mindlin R., Eshel N. (1968). On First Strain-Gradient Theories in Linear Elasticity. Int. J. Solids Struct..

[4] Maugin G. (1990). Internal Variables and Dissipative Structures. J. Non-Equilib. Thermodyn..

[5] Broese C., Tsakmakis C., Beskos D. (2016). Mindlin’s micro-structural and gradient elasticities and their thermodynamics. J. Elast..

[6] Dunn J., Serrin J. (1985). On the Thermodynamics of interstitial working. Arch. Ration. Mech. Anal..

[7] Auffray N., Dell’Isola F., Eremeyev V., Madeo A., Rosi G. (2015). Analytical continuum mechanics à la Hamilton–Piola least action principle for second gradient continua and capillary fluids. Math. Mech. Solids.

[8] Auffray N., Dell’Isola F., Eremeyev V., Madeo A., Placidi L., Rosi G., dell’Isola F., Maier G., Perego U., Andreaus U., Esposito R., Forest S. (2014). Least action principle for second gradient continua and capillary fluids: A Lagrangian approach following Piola’s point of view. The Complete Works of Gabrio Piola: Volume I.

[9] Alber H.D., Hutter K., Tsakmakis C. (2016). Nonconventional Thermodynamics, Indeterminate Couple Stress Elasticity and Heat Conduction. Contin. Mech. Thermodyn..

[10] Broese C., Tsakmakis C., Beskos D. (2018). Gradient Elasticity Based on Laplacians of Stress and Strain. J. Elast..

[11] Dunn J., Serrin J. (1986). Interstitial working and a nonclassical Continuum Thermodynamics. New Perspectives in Thermodynamics.

[12] Gurtin M., Fried E., Anand L. (2010). The Mechanics and Thermodynamics of Continua.

[13] Coleman B., Noll W. (1963). The thermodynamics of elastic materials with heat conduction and viscosity. Arch. Ration. Mech. Anal..

[14] Coleman B. (1964). Thermodynamics of materials with memory. Arch. Ration. Mech. Anal..

[15] Maugin G. (2006). On the Thermomechanics of Continuous Media With Diffusion and/or Weak Nonlinearity. Arch. Appl. Mech..

[16] Ireman P., Nguyen Q.S. (2004). Using the Gradients of the Temperature and Internal Parameters in Continuum Thermodynamics. Comptes Rendus Mec..

[17] Polizzotto C. (2003). Gradient elasticity and nonstandard boundary conditions. Int. J. Solids Struct..

[18] Polizzotto C. (2011). A unified residual-based thermodynamic framework for strain gradient theories of plasticity. Int. J. Plast..

[19] Liebe T., Steinmann P. (2001). Theory and numerics of a thermodynamically consistent framework for geometrically linear gradient plasticity. Int. J. Numer. Meth. Eng..

[20] Liebe T., Steinmann P., Benallal A. (2001). Theoretical and computational aspects of a thermodynamically consistent framework for geometrically linear gradient damage. Comput. Methods Appl. Mech. Eng..

[21] Edelen D., Laws N. (1971). On the Thermodynamics of Systems with Nonlocality. Arch. Ration. Mech. Anal..

[22] Liu I.S. (2002). Continuum Mechanics.

[23] Green A., Naghdi P. (1991). A re-examination of the basic postulates of thermodynamics. Proc. R. Soc. Lond. A.

